# Blood and Urine Cadmium, Blood Pressure, and Hypertension: A Systematic Review and Meta-analysis

**DOI:** 10.1289/ehp.1002077

**Published:** 2010-08-17

**Authors:** Carolyn M. Gallagher, Jaymie R. Meliker

**Affiliations:** 1 Doctoral Program in Population Health and Clinical Outcomes Research and; 2 Department of Preventive Medicine, Stony Brook University Medical Center, Stony Brook, New York, USA;; 3 Graduate Program in Public Health, State University of New York at Stony Brook, Stony Brook, New York, USA

**Keywords:** blood, blood pressure, cadmium, hypertension, meta-analysis, smoking, systematic review, urine

## Abstract

**Background:**

Cadmium exposure has been inconsistently related to blood pressure.

**Objectives:**

We updated and reevaluated the evidence regarding the relationships of blood cadmium (BCd) and urine cadmium (UCd) with blood pressure (BP) and hypertension (HTN) in nonoccupationally exposed populations.

**Data sources and extraction:**

We searched PubMed and Web of Science for articles on BCd or UCd and BP or HTN in nonoccupationally exposed populations and extracted information from studies that provided sufficient data on population, smoking status, exposure, outcomes, and design.

**Data synthesis:**

Twelve articles met inclusion criteria: eight provided data adequate for comparison, and five reported enough data for meta-analysis. Individual studies reported significant positive associations between BCd and systolic BP (SBP) among nonsmoking women [β = 3.14 mmHg per 1 μg/L untransformed BCd; 95% confidence interval (CI), 0.14–6.14] and among premenopausal women (β = 4.83 mmHg per 1 nmol/L log-transformed BCd; 95% CI, 0.17–9.49), and between BCd and diastolic BP (DBP) among women (β = 1.78 mmHg comparing BCd in the 90th and 10th percentiles; 95% CI, 0.64–2.92) and among premenopausal women (β = 3.84 mmHg per 1 nmol/L log-transformed BCd; 95% CI, 0.86–6.82). Three meta-analyses, each of three studies, showed positive associations between BCd and SBP (*p* = 0.006) and DBP (*p* < 0.001) among women, with minimal heterogeneity (*I*^2^ = 3%), and a significant inverse association between UCd and HTN among men and women, with substantial heterogeneity (*I*^2^ = 80%).

**Conclusion:**

Our results suggest a positive association between BCd and BP among women; the results, however, are inconclusive because of the limited number of representative population-based studies of never-smokers. Associations between UCd and HTN suggest inverse relationships, but inconsistent outcome definitions limit interpretation. We believe a longitudinal study is merited.

Hypertension (HTN) and smoking are established risk factors for cardiovascular disease (U.S. Department of Health and Human Services 2000), the leading cause of death worldwide (World Health Organization 2009). The etiology of essential HTN, however, is unknown (Carretero and Oparil 2000), but cadmium exposure has been inconsistently associated with blood pressure (BP). For example, Järup and Akesson (2009) recently reviewed the literature on cadmium and health effects and identified single-study–reported associations between cadmium and cardiovascular effects other than HTN. More than 10 years earlier, Nakagawa and Nishijo (1996) conducted a review of cadmium exposure and HTN and found that, although general population studies had reported positive associations of blood cadmium (BCd) and urinary cadmium (UCd) with BP, inverse associations had been reported in studies of residents or workers with known environmental or occupational exposures. Nakagawa and Nishijo (1996) interpreted these differences as an effect of low versus high exposures to cadmium, identified exposure misclassification as a limitation of studies conducted before the 1970s when cadmium measurements were semiquantitative, and also noted failure to account for the influence of smoking as a concern. Smoking is associated with increased cadmium levels because cigarettes contain cadmium taken up by the tobacco plant [Agency for Toxic Substances and Disease Registry (ATSDR) 2008]. Smokers have approximately twice the cadmium body burden of nonsmokers (ATSDR 2008). In nonsmokers, however, food is the primary source of exposure (ATSDR 2008). Nakagawa and Nishijo (1996) concluded that additional studies that control for smoking are needed, and several new studies that separated smokers from nonsmokers have been published since their review.

Since 1989, advancements in the technology to analyze BCd and UCd have improved the reliability of human exposure measures (Tsalev 1995); however, the use of these biomarkers has been inconsistent across epidemiological studies of HTN and BP. UCd is a biomarker for lifetime cadmium exposure among people with lower, nonoccupational exposures because, in the absence of episodes of high-level exposure, cadmium-binding sites, primarily in the kidney and liver, are not saturated, and UCd increases in proportion to the amount of cadmium stored in the body (Dillon and Ho 1991). UCd, however, can also reflect recent exposure (ATSDR 2008). BCd is a biomarker of recent exposure, with a half-life of 3–4 months, and is considered a biomarker for longer term exposure that reflects accumulation in the blood from body stores over a 10-year period (Järup et al. 1998). A greater percentage of inhaled rather than ingested cadmium is absorbed into the bloodstream (Järup et al. 1983, 1998). Thus, UCd and BCd levels may provide different information regarding the timing and source of exposure among smokers and nonsmokers.

The objectives of our systematic review and meta-analysis were to update and reevaluate the evidence regarding the relationships of BCd and UCd and BP and HTN and to discern the extent to which previously reported correlations may be associated with nonsmoking-related exposures, as indicated by BCd and UCd estimated effects in never-smokers.

## Materials and Methods

We conducted an electronic search using PubMed (National Library of Medicine; http://www.ncbi.nlm.nih.gov/sites/entrez) to locate all relevant articles that address BCd and/or UCd and BP in humans and smoking status. We used the following *a priori* inclusion criteria: UCd and BCd levels and systolic BP (SBP), diastolic BP (DBP), or HTN; the study population was not restricted to a specific disease, condition, or otherwise unique subset. We adjusted the statistical evaluation for smoking status, age, and sex; the difference in mean cadmium values between HTN cases and normotensive controls and/or associations between cadmium levels and BP and/or HTN were evaluated for statistical significance. We used cross-sectional, case–control, or cohort study design and conducted original analyses. To assess general population exposures, we excluded studies that specifically assessed occupationally exposed populations. We used the following medical subject heading (MeSH) terms—population, intervention and exposure, comparison, outcome, study design (Liberati et al. 2009): *a*) population: human AND adult AND NOT occupational exposure; *b*) biomarker of exposure: cadmium and administration and dosage, cadmium and adverse effects or cadmium and blood or cadmium and urine or cadmium and toxicity; *c*) comparisons: smoking status and sex (no MeSH terms specified); *d*) outcomes: BP or BP monitoring, ambulatory, or HTN; *e*) study designs: cross-sectional, case–control, cohort. We excluded studies that were limited to occupationally exposed populations (no MeSH terms specified).

Additionally, we conducted an electronic “bottom-up” search in the Web of Science (Thomson Reuters, New York, NY) to find articles that cite results of the PubMed literature search. Studies were limited to those published from 1989 to 2009 based on evidence of reliability of the technology to measure and analyze BCd and UCd (Tsalev 1995).

We developed a combined approach to weight the evidence of individual studies [see Supplemental Material, Table 1 (doi:10.1289/ehp.1002077)]. Study characteristics that merited higher weight-of-evidence (WOE) grades included separation by smoking status (i.e., either results were presented separately for smokers and never-smokers, or the population was restricted to never-smokers or nonsmokers), control for anti-HTN medication use, ambulatory or multiple BP measurements, analysis of both BCd and UCd biomarkers, or samples that represent general populations. In Table 1, the footnote gives the WOE codes that were used to qualitatively guide interpretation of the findings of the systematic review.

**Figure 1:**
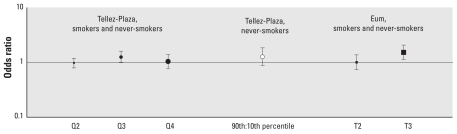
BCd dose–response comparisons: HTN ORs and 95% CIs. Tellez-Plaza et al. (2008): HTN = mean SBP ≥ 140, mean DBP ≥ 90 mmHg, self-report of a physician diagnosis or anti-HTN drug use; BCd (μg/L) quartiles: Q1 (ref), ≤ 0.20; Q2, 0.20–0.40; Q3, 0.40–0.70; Q4, ≥ 0.70. Eum et al. (2008): HTN = SBP ≥ 140, DBP ≥ 90 mmHg, or self-report of HTN; BCd (μg/L) tertiles: T1 (reference), 0.18–1.28; T2, 1.29–1.86; T3, 1.87–5.52. All data are for men and women combined. Sizes of different point estimate symbols for quartiles and tertiles reflect increasing BCd levels.

**Figure 2:**
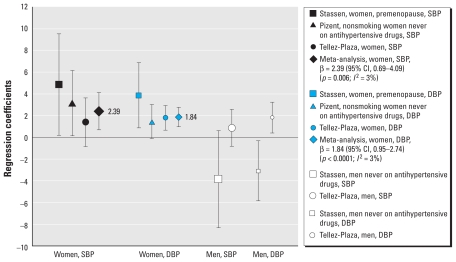
BCd: SBP and DBP, women and men separately (partial regression coefficients and 95% CIs).Staessen et al. (2000): BCd (nmol/L) = continuous log-transformed; 95% CI, coefficient ± 1.96 × SE. Pizent et al. (2001): BCd (μg/L) = continuous untransformed; 95% CI, coefficient ± 1.96 × SE. Tellez-Plaza et al. 2008: BCd (nmol/L), 90th to 10th percentile. Size of point estimate symbols varies to identify different

**Figure 3:**
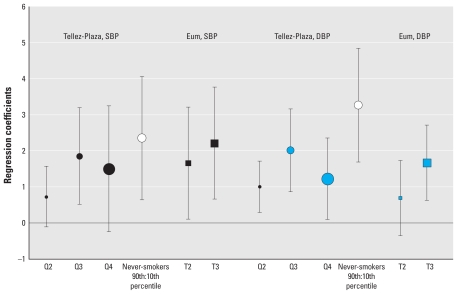
BCd dose–response comparisons: SBP and DBP (partial regression coefficients and 95% CIs) Tellez-Plaza et al. (2008): BCd (μg/L) quartiles: Q1 (reference), ≤ 0.20; Q2, 0.20–0.40; Q3, 0.40–0.70; Q4, ≥ 0.70. Eum et al. (2008): BCd (μg/L) tertiles: T1 (reference), 0.18–1.28; T2, 1.29–1.86; T3, 1.87–5.52. All data are for men and women combined. Sizes of different point estimate symbols for quartiles and tertiles reflect increasing BCd levels.

**Figure 4:**
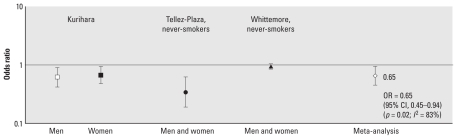
UCd and HTN (ORs and 95% CIs). Kurihara et al. (2004): HTN = SBP ≥ 140 or DBP > 90 mmHg, UCd (μg/g) = 84% upper cut-off level is 4.47 μg/g creatinine for men and 6.67 μg/g for women; spot urine sample, creatinine adjusted. Tellez-Plaza et al. (2008): HTN = mean SBP ≥ 140, mean DBP ≥ 90 mmHg, self-report of a physician diagnosis, or anti-HTN drug use; UCd (nmol/L) = 90th:10th percentile; spot urine sample, statistical model adjusted for creatinine. Whittemore et al. (1991): HTN = anti-HTN drug use, UCd (μg/L) = continuous untransformed; spot urine sample, specific gravity adjusted. Size of different point estimate symbols varies to identify different studies without quantitative or qualitative ranking.

**Figure 5:**
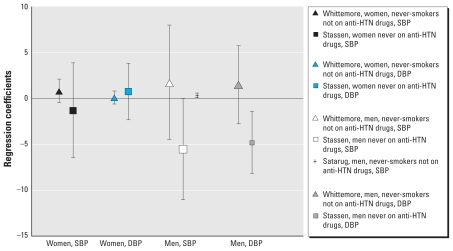
UCd: SBP and DBP, women and men separately (partial regression coefficients and 95% CIs). Whittemore et al. (1991): spot UCd (μg/L) adjusted for specific gravity, untransformed; 95% CI visually estimated from published graphs. Staessen et al. (2000): 24-hr UCd (nmol) log transformed; 95% CIs calculated as 1.96*SE. Satarug et al. (2005): 3-hr UCd (nanograms) log transformed; 95% CIs estimated from coefficient and *t*-value. Size of different point estimates varies to identify different studies without quantitative or qualitative ranking.

Findings of studies that reported multivariate-adjusted measures of association and 95% confidence intervals (CIs), and/or standard errors (SEs) or *t*-values, are presented in Figures 1–5. In the absence of reported CIs (Pizent et al. 2001; Satarug et al. 2005; Staessen et al. 2000; Whittemore et al. 1991), we calculated 95% CIs as 1.96 × SE and thus represent approximate intervals. Unreported SEs (Satarug et al. 2005) were calculated by dividing the reported coefficient by the reported *t*-value (Rosner 2006). For results presented in the original article in graph format only (i.e., findings for never-smokers reported by Whittemore et al. 1991), values for estimates and 95% CIs were visually approximated. Mean BCd and UCd values originally reported in nanomoles per liter were converted to micrograms per liter by dividing by 8.897, and creatinine-adjusted UCd values originally reported as nanomoles per millimole creatinine were converted to micrograms per gram creatinine by dividing by 1.006 (Tellez-Plaza 2008). Interpretations of statistical significance are based on an alpha level ≤ 0.05.

Because two studies are a suggested minimum requirement for a systematic review to include a meta-analysis (Littell et al. 2008), we required at least three studies with comparable exposure and outcome measures. Meta-analysis was conducted using random effects models and inverse variance methods to weight effect estimates. Random effects models were used to account for variation among the studies (Littell et al. 2008). Inverse variance methods were used to give greater weight to studies characterized by greater precision, that is, relatively narrow CIs (Littell et al. 2008). Meta-analysis was performed using Review Manager 5.0 (RevMan; Cochrane Collaboration, Copenhagen, Denmark, http://ims.cochrane.org/revman).

## Results

### Literature search

Electronic search results yielded a total of 33 citations; of these only 12 met the inclusion criteria [see Supplemental Material, Table 2 (doi:10.1289/ehp.1002077) for citations of excluded articles and the reasons for exclusion].

#### Large representative population-based samples, stratified by smoking status

Tellez-Plaza et al. (2008) analyzed data from the 1999–2004 National Health and Nutrition Examination Survey (NHANES; *n* = 10,991), and Whittemore et al. (1991) analyzed data from the 1976–1988 NHANES II (*n* = 960); both were cross-sectional studies (Table 1). Tellez-Plaza et al. (2008) defined HTN as mean SBP ≥ 140 mmHg, a mean DBP ≥ 90 mmHg, a self-reported physician diagnosis, or the use of medication for HTN; Whittemore et al. (1991) defined HTN by anti-HTN drug use only. Exposure measures included spot urine samples for both studies; however, Tellez-Plaza et al. (2008) used multivariate adjustment for creatinine to adjust for urine dilution effects, whereas Whittemore et al. (1991) directly adjusted UCd measurements for specific gravity. Tellez-Plaza et al. (2008) also estimated associations with BCd. Whittemore et al. (1991) estimated associations with continuous, untransformed UCd measures, and Tellez-Plaza et al. (2008) estimated associations for cadmium quartiles (relative to the lowest quartile) and for cadmium levels at or above the 90th percentile compared with cadmium at or below the 10th percentile, in addition to estimating associations with log-transformed continuous biomarker measures. The NHANES II database used by Whittemore et al. (1991) lacked appropriate sample weights for the subsample with cadmium measurements. Thus, *p*-values and CIs were calculated based on the assumption that this subsample is a simple random sample of the U.S. population. Tellez-Plaza et al. (2008) adjusted for HTN medication use in multivariate analyses but did not exclude treated HTN subjects from the analysis of never-smokers; in contrast, Whittemore et al. (1991) conducted two sets of analyses: One included all subjects and statistically adjusted for current hypertensive medication use, and the second set excluded subjects who had been treated for HTN.

#### Small studies, limited to nonsmokers

These four studies ranged in sample size from 53–267 subjects. Outcome measures included continuous SBP and DBP (Pizent et al. 2001; Satarug et al. 2005); dichotomous SBP and/or DBP, that is, SBP > 140 mmHg and/or DBP > 90 mmHg (Vivoli et al. 1989); mean SBP (Satarug et al. 2005); and mean arterial pressure (Lin et al. 1995). Exposure measures included 3-hr log-transformed UCd (Satarug et al. 2005), mean creatinine-adjusted spot UCd (Vivoli et al. 1989), and untransformed BCd (Lin et al. 1995; Pizent et al. 2001). The three cross-sectional studies were limited to nonsmokers (Lin et al. 1995; Pizent et al. 2001; Satarug et al. 2005), and the one case–control study matched cases and controls for smoking status (Vivoli et al. 1989). Treated HTN subjects were excluded from all four studies. Study populations were urban (Satarug et al. 2005) and rural (Pizent et al. 2001), clinic recruited (Lin et al. 1995), and occupation specific (Vivoli et al. 1989). Findings from Lin et al. (1995) are not depicted in graph format because the outcome measure (i.e., mean arterial BP) was not comparable with those of the other studies. Additionally, findings from Vivoli et al. (1989) are not plotted because this study analyzed the difference in mean cadmium between cases and controls and did not report comparable measures of association.

#### Large studies, not limited to nonsmokers

These three cross-sectional studies used the following number of subjects: 2,853 (Kurihara et al. 2004), 1,902 (Eum et al. 2008), and 1,223 (Menditto et al. 1998). Outcome measures included categorical measures of HTN, that is, SBP ≥ 140 mmHg and/or DBP > 90 mmHg or taking anti-HTN drugs (Kurihara et al. 2004) or SBP ≥ 140 mmHg or DBP ≥ 90 mmHg or self-reported HTN in medical examination (Eum et al. 2008); continuous SBP and DBP (Eum et al. 2008; Menditto et al. 1998); and mean BP, that is, DBP + pulse pressure ÷ 3 (Eum et al. 2008) and DBP + ^1^/_3_ × (SBP – DBP) (Menditto et al. 1998). Exposure measures included BCd tertiles (0.18–1.28 μg/L, 1.29–1.86 μg/L, 1.87–5.52 μg/L (Eum et al. 2008); 84% upper cutoff dichotomized BCd and UCd, that is, geometric means × geometric SDs: UCd, men, 1.8 × 2.5 = 4.5 μg/g; UCd, women, 2.4 × 2.8 = 6.72 μg/g; BCd, men, 2.2 × 1.9 = 4.18 μg/L; BCd, women, 2.3 × 1.8 = 4.14 μg/L (Kurihara et al. 2004); and continuous log-transformed BCd (Menditto et al. 1998). Kurihara et al. (2004) used multivariable analysis to control for smoking status but did not separate former smokers from nonsmokers. Eum et al. (2008) controlled for former, current, and never-smokers, and Menditto et al. (1998) controlled for number of cigarettes smoked per day. Each of these studies statistically adjusted for smokers and nonsmokers but did not present the results separately for these two groups. Eum et al. (2008) ran separate regression models for low (< 0.95 mg/dL), medium (≥ 0.95 and < 1.05 mg/dL), and high (≥ 1.05 mg/dL) serum creatinine to adjust for renal dysfunction, and Kurihara et al. (2004) adjusted for β-2-microglobulin, which is a measure of tubular renal dysfunction. The study conducted by Menditto et al. (1998) was unique among this group of studies because they excluded treated hypertensive subjects; however, they did not report measures of association.

#### Small studies, not limited to nonsmokers

These studies ranged in sample size from 154 to 692 subjects. Outcome measures included SBP and DBP (Schutte et al. 2008; Staessen et al. 2000; Telisman et al. 2001) and 24-hr ambulatory SBP and DBP (Staessen et al. 2000). Exposure measures included 24-hr log-transformed UCd (Schutte et al. 2008; Staessen et al. 2000) and log-transformed BCd (Schutte et al. 2008; Staessen et al. 2000; Telisman et al. 2001). Staessen et al. (2000) conducted a combined cross-sectional and prospective study of 692 residents of two rural areas in Belgium, one with known environmental exposures to cadmium from zinc smelters. The study period included the years 1985–1989 and 1991–1995 for the same participants (less those lost to follow-up) after interventions to reduce cadmium exposure. The Schutte et al. (2008) analysis evaluated cross-sectional data from a sample of 557 subjects from this same study restricted to the years 1991–1994 and included 26 occupationally exposed men. A case–control study (Telisman et al. 2001) restricted participants to 154 nonoccupationally exposed men; however, measures of association were not presented.

### Comparison of multivariate adjusted estimated effects

Results from eight studies provided adequate data to compare estimated effects. Results from five studies provided sufficient data for meta-analysis, using three studies for each analysis, with one study used in two meta-analyses of different exposures.

#### BCd and HTN

Figure 1 presents the estimated dose–response effects of BCd on HTN. In multivariable analysis that adjusted for smoking status and use of anti-HTN medications, Tellez-Plaza et al. (2008) estimated associations between HTN and BCd levels categorized by quartiles, with the first quartile used as the reference group (BCd ≤ 0.20 μg/L); quartile 2 = 0.20–0.40 μg/L; quartile 3 = 0.40–0.70 μg/L; and quartile 4 = > 0.70 μg/L. Relative to the first quartile (819 cases and 1,689 noncases), subjects in the third quartile (1,452 cases and 1,369 noncases) were 25% more likely to be hypertensive [odds ratio (OR) = 1.25; 95% CI, 0.87–1.81], but HTN was not associated with exposures in the second and fourth quartiles. Additionally, the authors compared the 90th and 10th percentiles in never-smokers (*n* = 5,486); the nonsignificant effect estimate (OR = 1.14; 95% CI, 0.89–1.45) was equivalent to that of the third quartile. Eum et al. (2008) categorized BCd levels into tertiles, with tertile 1 (reference group) ranging from 0.18 to 1.28 μg/L, tertile 2 from 1.29 to 1.86 μg/L, and tertile 3 from 1.87 to 5.52 μg/L. Subjects in the highest tertile were 52% more likely to have HTN than were those in the lowest tertile of BCd (OR = 1.52; 95% CI, 1.13–2.05).

#### BCd, and SBP and DBP

Figure 2 shows the relationships between BCd and SBP and DBP in men and women separately. Tellez-Plaza et al. (2008) reported that, in men, BCd (nmol/L) in the 90th relative to the 10th percentile was significantly associated with DBP (β = 1.81 mmHg; 95% CI, 0.40–3.22); this relationship, however, was not significant for SBP. In contrast, Staessen et al. (2000) reported inverse associations of log-transformed BCd (nmol/L) with SBP and DBP among men never on anti-HTN medications; however, this inverse relationship was only significant for DBP (β = −3.10 mmHg; 95% CI, −5.86 to −0.34). Because results from a third study were not available, meta-analysis was not performed using these findings for men.

Results were available from three studies that evaluated the relation between BCd and SBP among women, so we conducted a meta-analysis. Statistically significant positive associations were reported by Pizent et al. (2001) for a one-unit increase in untransformed BCd (micrograms per liter) among nonsmoking women (β = 3.14 mmHg; 95% CI, 0.14–6.14) and by Staessen et al. (2000) for a one-unit increase in log transformed BCd (nanomoles per liter) among premenopausal women (β = 4.83 mmHg; 95% CI, 0.17–9.49). Tellez-Plaza et al. (2008) also reported positive, although insignificant, associations between BCd (nanomoles per liter), comparing 90th and 10th percentiles and SBP among women (β = 1.40 mmHg; 95% CI, −0.81 to 3.61). Overall estimated effects were significantly positive (β = 2.39 mmHg; 95% CI, 0.69–4.09; *p* = 0.006), with minimal heterogeneity (*I*^2^ = 3%).

All three studies showed positive relationships between BCd and DBP, with similar effect estimates, among women; however, only the findings of Staessen et al. (2000) and Tellez-Plaza et al. (2008) showed statistical significance (respectively: β = 3.84 mmHg; 95% CI, 0.86–6.82; β = 1.78; 95% CI, 0.64–2.92). The effect estimate for nonsmoking women found by Pizent et al. (2001) was similar to that found by Tellez-Plaza et al. (2008) for all women (β = 1.40 mmHg; 95% CI, −0.15 to 2.95). As in the meta-analysis for BCd and SBP, overall associations for BCd and DBP were significantly positive (β = 1.84 mmHg; 95% CI, 0.95–2.74; *p* < 0.0001), with minimal heterogeneity (*I*^2^ = 3%).

#### BCd dose response

In Figure 3, we compared BCd associations with SBP and DBP by levels of exposure. Among never-smokers, BCd levels in the 90th percentile were significantly associated with elevated SBP (β = 2.35 mmHg; 95% CI, 0.64–4.05) and DBP (β = 3.25 mmHg; 95% CI, 1.69–4.84) relative to the 10th percentile (Tellez-Plaza et al. 2008). In the smoking-adjusted analysis, the third quartile of BCd exposure relative to the lowest level showed a larger estimated effect (SBP: β = 1.85 mmHg; 95% CI, 0.52–3.19; DBP: β = 2.01 mmHg; 95% CI, 0.86–3.15) than did the second quartile; however, for both SBP and DBP, the effect estimate for the fourth quartile was attenuated relative to that of the third (Tellez-Plaza et al. 2008). For SBP, Eum et al. (2008) reported a positive association with the second tertile of BCd compared with the reference group (β = 1.651 mmHg; 95% CI, 0.099–3.203) and a slightly stronger association with BCd exposures in the third tertile (β = 2.204 mmHg; 95% CI, 0.649–3.760) relative to the first quartile. For DBP, only the third tertile reached statistical significance (β = 1.671 mmHg; 95% CI, 0.626–2.716). Comparisons of second and third levels across these two studies of lower population mean BCd (0.42 μg/L; Tellez-Plaza et al. 2008) and higher population mean BCd (1.67 μg/L; Eum et al. 2008) suggest a positive dose response.

#### UCd and HTN

Figure 4 presents study findings regarding the association between UCd and HTN. Tellez-Plaza et al. (2008) used a dichotomous measure of UCd obtained from spot urine samples, compared the 90th and 10th percentiles, and used multivariate adjustment to statistically adjust for urine creatinine; in contrast, Kurihara et al. (2004) used an 84% cutoff (4.5 μg/g for men and 6.72 μg/g for women) and directly adjusted UCd for urine creatinine. Whittemore et al. (1991) also measured cadmium from spot urine samples but adjusted for specific gravity. They used anti-HTN drug use to define HTN, whereas Kurihara et al. (2004) used standard BP cutoff measures, and Tellez-Plaza et al. (2008) incorporated both definitions, as well as self-report of a physician diagnosis of HTN. Despite these methodological differences, inverse relationships were found between UCd and HTN (HTN) among men and among women. ORs varied considerably across studies, with ORs of 0.62 for men and 0.67 for women in the Kurihara et al. (2004) study, and ORs of 0.34 and 0.94 for never-smoking men and women in the Tellez-Plaza et al. (2008) and Whittemore et al. (1991) studies, respectively. Meta-analysis of results from these three studies showed UCd to be significantly negatively associated with HTN (OR = 0.65; 95% CI, 0.45–0.94; *p* = 0.02); however, heterogeneity was substantial (*I*^2^ = 83%).

#### UCd, and SBP and DBP

Figure 5 presents partial regression coefficients (adjusted for covariates in multivariable analysis) and 95% CIs for the relationship of UCd with SBP and DBP, evaluated separately for women and men. Statistically significant inverse relationships for a one-unit increase in log-transformed 24-hr UCd (nanomoles/liter) with SBP (β = −5.55 mmHg; 95% CI, −11.04 to −0.06) and DBP (β = −4.80 mmHg; 95% CI, −8.19 to −1.41) were reported for men who were never on anti-HTN drugs (Staessen et al. 2000). Although Whittemore et al. (1991) reported positive relationships of untransformed UCd (μg/L) with SBP and DBP among both never-smoking men and women, associations were not statistically significant. Satarug et al. (2005) observed a statistically significant positive relationship of log-transformed 3-hr UCd (nanomolar) with SBP (β = 0.31 mmHg; 95% CI, 0.05–0.57) among nonsmoking men who were not on anti-HTN medications; however, the estimated effect size was small, and findings were null for women. Overall, these results are inconsistent, which may be attributable, in part, to different units of exposure measures, variations in sample sizes, and differences in smoking status and anti-HTN drug use.

## Discussion

### Synthesis of findings using a causal inference framework

Causal inference criteria provide a framework useful to interpret the strength and limitations of the evidence regarding an association between BCd and/or UCd with BP. Hill (1965) and contemporaries (Kundi 2006; Rothman and Poole 2007) caution against using epidemiological causal inference criteria as a checklist. Noting these cautionary concerns, it is informative to qualitatively group these criteria as follows: strength of association, consistency among studies, and temporality; and dose response, epidemiological coherence, and biological plausibility.

#### Strength of association, consistency, and temporality

Positive associations between BCd with elevated SBP and DBP were found among nonsmokers (Pizent et al. 2001) and never-smokers (Tellez-Plaza et al. 2008). Statistically significant relationships between BCd, SBP and DBP among nonsmokers and never-smokers are interpreted as providing stronger evidence than associations from smoking-adjusted studies because the effects of current and ever-smoking, respectively, are removed rather than statistically adjusted for. Meta-analysis supported strength of association, and the relationship between BCd and BP was evident across three studies of women (Pizent et al. 2001; Staessen et al. 2000; Tellez-Plaza et al. 2008), regardless of smoking adjustment or stratification methods. In the only prospective study, Staessen et al. (2000) found that BCd was positively related to SBP and DBP in premenopausal women. Moreover, a longitudinal decrease in BCd was documented after environmental remediation, and decreased BCd was associated with decreased DBP in women (Staessen et al. 2000). Because BCd is more influenced by recent exposure, and SBP and DBP are concurrent measures, the evidence suggests a temporal relationship between BCd and recent effects. BCd may also reflect accumulation of cadmium with age; however, all studies adjusted for or matched on age.

BCd was less consistently associated with HTN. This may be due to the disparate definitions of HTN. Studies similar in terms of adjustment for measures of renal dysfunction and populations with relatively high BCd levels reported discrepant findings; specifically, Eum et al. (2008) reported positive associations between BCd, and BP and HTN in a sample with a geometric mean > 2.0 μg/L, whereas Kurihara et al. (2004) reported no association between BCd and HTN in a sample with similarly high BCd (1.67 μg/L). BCd means for both of these samples were greater than that of the NHANES sample (0.42 μg/L) (Tellez-Plaza et al. 2008), and BCd was positively associated with BP in this low-exposure population, as well as in the Eum et al. (2008) study of a high-exposure population. Thus, the results of the present review do not support Nakagawa and Nishijo’s (1996) conclusions that general populations with low exposures show positive associations between cadmium and BP, whereas populations with kidney dysfunction and high exposures show inverse associations. Of note, the only study reviewed in both the present and original review was the Staessen et al. (2000) study; however, Nakagawa and Nishijo (1996) referenced earlier versions (Staessen et al. 1984, 1991) and thus did not include the more recent findings of a positive association between BCd and BP in women (Staessen et al. 2000).

Several studies showed an inverse association between UCd, a biomarker of long-term exposure, and HTN. This inverse relationship was evident in both high- and low-exposure populations, so again, this does not support the earlier systematic review’s interpretation that inverse associations between cadmium and BP are characteristic of populations with higher exposures and associated renal dysfunction (Nakagawa and Nishijo 1996). Specifically, both the Tellez-Plaza et al. (2008) study of a low-exposure population (mean BCd = 0.42 μg/L; mean UCd = 0.28 μg/L) and the Kurihara et al. (2004) study of a high-exposure population (BCd geometric mean, 2.2–2.3 μg/L; UCd geometric mean 1.8–2.4 μg/g creatinine) found statistically significant inverse relationships between UCd and HTN. Staessen et al. (2000) evaluated SBP and DBP averaged over 15 readings taken during the period 1985–1995; this time-integrated analysis also showed an inverse relationship between UCd and long-term DBP in men.

A limitation common to all studies, and thus to the meta-analysis of the relation between UCd and HTN, is that the outcome of HTN was not consistently defined across studies. Although meta-analysis findings support an inverse relationship, the finding of substantial heterogeneity might reflect outcome misclassification. Thus, although causal inference criteria support the interpretation of a positive association between BCd and higher SBP and DBP, the relationship between UCd, and BP and HTN remains uncertain.

#### Dose response, epidemiologic coherence, and biologic plausibility

Dose–response analyses of BCd tertiles and quartiles were not restricted to never-smokers, so interpretations regarding cadmium’s exposure–response effects independent of smoking are limited. It is notable in the Tellez-Plaza et al. (2008) study, however, that for the outcomes of SBP and DBP, never-smokers show the largest effect estimates when comparing the 90th and 10th percentiles of BCd exposures and that, in the smoking-adjusted analysis of dose response in this same study, the fourth quartile of cadmium exposure shows a smaller effect estimate compared with the third quartile. Some studies show that smokers have lower BP than do nonsmokers (Green et al. 1986; Primatesta et al. 2001; Stolarz et al. 2003), and Lee (2008) found that smoking was a risk factor for masked HTN, that is, normal clinic BP but elevated ambulatory BP, suggesting that effect estimates in the upper range of cadmium exposure may be confounded by cigarette smoking. This hypothesis warrants investigation.

Based upon animal and *in vitro* studies, cadmium may increase BP through vascular effects. A hypothesized mechanism of action (MOA) for cadmium in humans is inhibition of endothelial nitric oxide synthase protein in blood vessels, which suppresses acetylcholine-induced vascular relaxation to induce HTN (Yoopan et al. 2008). On the other hand, serum cotinine, a metabolite of nicotine, has been inversely related to BP in smokers (Benowitz and Sharp 1989), and Ghasemi et al. (2010) reported a significantly positive correlation between serum nitric oxide and the number of cigarettes smoked per day, suggesting a possible MOA for how smoking might confound the relationship between cadmium and BP.

The inverse relationships observed between UCd and BP raise the question of whether cadmium might have depressor effects. Experimental findings suggest that cadmium binds to calcium-binding sites on the regulatory protein calmodulin, and like calcium, cadmium can increase dopamine synthesis in the brain that lowers BP (Sutoo and Akiyama 2000). Further research is merited to investigate this hypothesized MOA in humans.

HTN is a disease of differential physiological characterization. Approximately one-fourth of HTN subjects, particularly those with renovascular HTN, show high levels of angiotensin II, a vasoconstrictor (Malpas 2010). Angiotensin II receptor binding sites are located in the brain at sites involved with sympathetic nerve activity via baroreflex regulation (Malpas 2010). Puri and Saha (2003) found that in rats cadmium inhibited angiotensin-converting enzyme (ACE) at low, medium, and high doses without a dose–response effect yet paradoxically induced HTN; they postulated that cadmium’s vascular effects predominated over its central effects in HTN rats. Although cadmium’s central versus vascular effects in humans are unknown, it has been shown that the ACE inhibitor valsartan is more effective in preventing cardiac failure in HTN men than in HTN women (Zanchetti et al. 2006). In light of meta-analysis findings of an association between BCd and elevated BP in women, perhaps future research into cadmium’s mechanisms of action may lead to improved gender-specific therapeutic interventions.

Staessen et al. (2000) found an inverse association between BCd and BP in men never on anti-HTN drugs. This finding and the meta-analysis finding of UCd’s inverse association with HTN, yet UCd’s positive associations with heart failure (Peters et al. 2010), seem counterintuitive, because HTN is an established risk factor for cardiovascular disease. In as many as 33% of HTN heart disease patients, however, heart failure is unrecognized because as this condition develops, the left ventricle becomes too weak to raise DBP (Riaz 2010). Further, masked HTN is prevalent in 10–20% of the adult population (O’Brien 2008). The extent to which undiagnosed and untreated HTN disease is associated with cadmium exposure has not been evaluated.

### Methodological critique of individual studies

Cross-sectional analysis and inadequate specification of the duration of HTN limit temporal interpretations. Misclassification bias may result from the inconsistent measurement of HTN across studies. Even the measurement of BP may be biased by the phenomenon of masked HTN, which has been associated with cardiac and arterial target organ damage comparable with that of sustained HTN (Kotsis et al. 2008). Hypertensive heart failure is of even greater prevalence (Riaz 2010), and thus, nonmeasurement may be an additional source of outcome misclassification.

Sample selection considerations and exposure measurement error are additional limitations in these studies. Staessen et al. (2000) included men with known occupational exposures, as did Schutte et al. (2008), thus limiting interpretations of findings in men. Further, industrial exposures to cadmium emissions may have uniquely influenced dietary cadmium intake for subjects who consumed food grown in cadmium-contaminated soil. Of the six studies that separated smokers from nonsmokers, the four smaller studies used specific samples that limited generalizability of findings, and the Whittemore et al. (1991) study was not a probability sample. Treated HTN subjects were either analyzed separately or excluded in all smoking-stratified studies except the Tellez-Plaza et al. (2008) study. Further, the use of spot urine samples in the Tellez-Plaza et al. (2008), Whittemore et al. (1991), Vivoli et al. (1989), and Kurihara et al. (2004) studies may limit the accuracy of exposure assessment due to variable urinary dilution effects throughout the day (Barr et al. 2005). Urine specific gravity and creatinine correction were used to address this limitation, however, and Berlin et al. (1985) reported a correlation between cadmium levels measured in spot and 24-hr samples from occupationally exposed subjects.

### Limitations of meta-analysis

The small number of studies precluded quantitative bias assessment, as well as meta-analysis of the relation between BCd with SBP and DBP among men. Further, Menditto et al. (1998) and Kurihara et al. (2004) did not report statistics for null findings regarding the relation between BCd and BP, so meta-analysis may be subject to positive reporting bias. On the other hand, Lin et al. (1995) and Vivoli et al. (1989) found positive relationships between BCd and BP but did not report comparable measures of association, which may have subjected the meta-analysis to negative reporting bias. Meta-analysis of SBP and DBP used both continuous (Pizent et al. 2001; Staessen et al. 2000) and 90:10th percentile exposure measures (Tellez-Plaza et al. 2008). Similarly, meta-analysis of HTN used both continuous (Whittemore et al. 1991) and high:low UCd exposure measures (Kurihara et al. 2004; Tellez-Plaza et al. 2008). Further, units of measure varied across studies. Thus, there were substantial differences in exposure measures that limited interstudy comparisons of effect estimates.

## Conclusion and Recommendations

The body of evidence relating BCd to BP suggests a positive relationship, especially in females, but in the absence of dose–response gradients in never-smokers is inconclusive. The inverse relationships between UCd and BP reported in the meta-analysis lack strong mechanistic support. Our findings offer new insights, however, because these paradoxical relationships were evident in both high- and low-exposure populations, as indicated by mean population cadmium exposure levels, and thus contradict earlier assumptions that this inverse association only reflected higher cadmium exposures. In light of this review’s evidence of an association between BCd and higher BP, an established risk factor for cardiovascular disease, and recent evidence of a prospective association between long-term cadmium exposure and cardiovascular mortality (Menke et al. 2009), cadmium merits further epidemiologic inquiry. The European Food Safety Authority (2009) recognized that cadmium has been associated with myocardial infarction (Everett and Frithsen 2008) and alterations in cardiovascular function (Schutte et al. 2008). More rigorous investigation of both short- and longer-term effects of nonsmoking cadmium exposures may shed insights regarding susceptibility to HTN and cardiovascular disease by identifying cadmium dose–response relationships over time.

This line of research would benefit from both physiological studies of cadmium’s MOA, and longitudinal epidemiological studies of never-smoking, general populations (i.e., non-occupationally and nonindustrially exposed) to evaluate the relationships among BCd and UCd, and SBP, DBP, and sustained HTN. Sufficient power would be needed to examine effects in the never-smoking general population, with subset analyses by gender,

A longitudinal study would help tease out temporally relevant influences, such as menopausal status and hormonal effects. Cadmium has been shown to suppress progesterone production (Paksy et al. 1997) and has also been associated with increased serum levels of follicle-stimulating hormone (Gallagher et al. 2010). Insights regarding gender differences in cadmium toxicokinetics may be gained by measuring iron levels, because iron competes with cadmium for binding sites on the metal transporter divalent metal transporter 1 (DMT1) (Nishijo et al. 2004). Because cadmium has been associated with peripheral arterial disease (PAD) (Navas-Acien et al. 2004), and zinc and UCd were inversely associated in patients with PAD (Tsai et al. 2004), zinc intake also merits consideration. Further, Guallar et al. (2006) found that BCd partially explained the relationship between elevated homocysteine levels and PAD. Methylenetetrahydrofolate reductase (MTHFR) is a key enzyme in homocysteine metabolism, and *MTHFR* gene polymorphisms were associated with essential HTN (Ilhan et al. 2008).

An increasing body of evidence suggests that cadmium is a risk factor for cardiovascular morbidity and mortality, as well as a contaminant of concern in our food supply (European Food Safety Authority 2009; Reuben 2010). Findings from this meta-analysis indicate a positive association between BCd and increased BP, particularly in women, and identify gaps in research regarding the association of cadmium exposure with HTN. Longitudinal studies are merited to evaluate the relationships of cadmium exposures with more rigorous measures of HTN; physiological indicators of cadmium’s central, cardiac, and vascular effects; hormonal nutritional factors; genetic susceptibilities; and cardiovascular disease among never-smokers.

## Figures and Tables

**Table 1:** Study characteristics, key findings, and WOE codes.

Study population	Exposure measure	Covariates	Outcome measure	Key findings^a^	Study design/WOE codes^b^	Reference
Large representative population-based samples, with smoking-stratified findings						
U.S. men and women ≥ 20 years of age; *n* = 10,991; mean BCd, 0.42 μg/L; UCd, 0.28 μg/L	BCd, spot UCd without direct dilution adjustment	Age, sex, race, education, cotinine, alcohol, BMI, menopause, anti-HTN drug use, blood lead, and urinary creatinine for UCd	HTN = mean SBP ≥ 140 mmHg, mean DBP ≥ 90 mmHg; self-reported physician diagnosis, or drug use; SBP, DBP, 3–4 measures	Never-smokers: BCd + SBP BCd + DBP BCd o HTN UCd – HTN	Cross-sectional (NHANES 1999–2004), cadmium-weighted sample: A A A/B B	Tellez-Plaza et al. 2008
U.S. men and women 20–74 years of age; mean UCd: men, 1.1 μg/L; women, 1.3 μg/L; *n* = 960	Spot UCd, adjusted for specific gravity	Age, sex, race, Quetelet’s index, family history HTN, and anti-HTN drug use; never-smokers exclude HTN drug use	HTN = anti-HTN drug use; SBP, DBP, three measures	Never-smokers: UCd o SBP UCd o DBP UCd o HTN	Cross-sectional (NHANES II 1978–1979), cadmium-unweighted sample: B A/B A B	Whittemore et al. 1991
Small studies, limited to nonsmokers						
Adults 16–60 years of age, near Bangkok, Thailand, no occupational exposures; *n* = 100 women and 53 men who were never-smokers; UCd mean: women, 3.5 nM/3; men, 2.7 nM/3	3-hr UCd	Age, BMI, urine lead; excluded subjects on anti-HTN drugs or any medication on a regular basis	SBP, DBP, MBP	Never-smokers: Men, UCd + SBP Women, UCd o SBP	Cross-sectional: B B A C	Satarug et al. 2005
Never-smoking women 31–77 years of age, from routine and HTN clinics, Taiwan, near cadmium-polluted area; mean BCd: no HTN (*n* = 24), 0.88 μg/L; untreated essential HTN (*n* = 24), 1.69 μg/L; nonessential HTN (*n* = 10), 0.92 μg/L	BCd, 24-hr UCd, creatinine adjusted	Age, BMI; excluded smokers and occupational exposure history, proteinuria, hematuria, low creatinine clearance	MBP, averaged from three different visits on three different days	BCd + MBP UCd + MBP	Cross-sectional: B B A C	Lin et al. 1995
“Peasant” women 40–85 years of age from rural Croatia, all nonsmokers (included former smokers); *n* = 267; median BCd, 0.6 μg/L	BCd	Area of residence (high vs. low calcium intake), age, alcohol, BMI, serum lead; excluded subjects on drugs that could affect BP	SBP, DBP	BCd + SBP BCd + DBP	Cross-sectional: B B A/B C	Pizent et al. 2001
Male bankers from Modena, Italy; mean age, 37–38 years; epidemiological screening: BCd mean hypertensive and normotensive, 0.58/0.44 μg/L; UCd (creatinine adjusted) mean hypertensive andnormotensive, 1.36/1.23 μg/g; *n* = 63 HTN and 63 non-HTN	BCd; spot UCd, creatinine adjusted	Matched for age, smoking, anthropometrics, work conditions; excluded taking drugs for HTN	SBP > 140 and/or DBP > 90; 2 readings	Mean BCd: cases, 0.41 μg/L; controls, 0.25 μg/L (*p* < 0.01); no significant difference in UCd between cases and controls	Case–control: A B A C	Vivoli et al. 1989
Large studies, not limited to nonsmokers						
Nonoccupationally exposed, ≥ 50 years of age, from three “unpolluted” rural areas, Japan; *n* = 1,140 men and 1,713 women; geometric mean BCd: men, 2.2 μg/L; women, 2.3 μg/L; geometric mean UCd (creatinine adjusted): men, 1.8 μg/g; women, 2.4 μg/g	BCd; spot UCd, creatinine adjusted	Age, smoking (nonsmoker, including ex-smoker, and current smokers) alcohol intake, serum creatinine; BMI, beta-2 microglobulin	HTN = SBP > 140 or DBP > 90 or taking anti-HTN drugs	BCd o HTN UCd – HTN	Cross-sectional: B C B/C C	Kurihara et al. 2004
Korean men and women ≥ 20 years of age; *n* = 958 men and 944 women; mean BCd, 1.67 μg/L	BCd; T1, 0.18–1.28 μg/L (ref); T2, 1.29–1.86 μg/L; T3, 1.87–5.52 μg/L	Age, sex, education, smoker (never, ex, current), alcohol intake, BMI, self-reported HTN, family HTN, blood lead; also stratified by serum creatinine	HTN = SBP ≥ 140 or DBP ≥ 90 or self-report; SBP, DBP; MBP = DBP + pulse pressure/3	BCd T2 + SBP BCd T3 + SBP BCd T3 + DBP BCd T3 + MBP BCd T3 + HTN Effect of BCd on BP strengthened with renal dysfunction	Cross-sectional (KHANES 2005): B A/B B/C B	Eum et al. 2008
Men 55–75 years of age from Rome, Italy; excluded treated HTN subjects; *n* = 1,223; mean BCd, 0.62 μg/L	BCd	Age, alcohol consumption, number of cigarettes/day, BMI, HDL cholesterol, non-HDL cholesterol, serum lead, heart rate, driving min/day, skin-fold thickness	SBP, DBP; MBP = DBP + ^1^/_3_(SBP − DBP)	BCd o SBP BCd o DBP BCd o MBP	Cross-sectional (New Risk Factors Project, June 1989 to December 1990): B B A/B B	Menditto et al. 1998
Small studies, not limited to nonsmokers						
Men and women ≥ 20 years of age; 2 rural areas in Belgium, one near three zinc smelters; arithmetic mean BCd for high and low exposure areas, 0.98/0.08 μg/L; geometric mean 24-hr UCd for high and low exposure areas, 9.8/7.1 nmol/24-hr urine; *n* = 557, including 41 men and 37 women smokers, 26 occupationally exposed men	BCd, 24-hr UCd	Sex, age, BMI, γ-glutamyl-transferase, blood glucose, current smoker versus nonsmoker, anti-HTN treatment, total cholesterol, HDL	SBP, DBP; MBP = DBP + 1/3 pulse pressure; average of five consecutive readings	UCd – SBP BCd o SBP BCd o DBP BCd o MBP	Cross-sectional (1991–1994): A C B/C B	Schutte et al. 2008
Same as above, except included time period before interventions to reduce exposure levels; baseline geometric mean BCd for men and for women, 1.26/1.23 μg/L; baseline geometric mean 24 hr UCd for men and for women, 11.8/8.8 nmol/24 hr; *n* = 336 men and 356 women	BCd, 24-hr UCd	SBP, DBP; average of five consecutive readings; time-integrated analysis of SBP and DBP averaged over fifteen readings, 1985–1995; 24-hr ambulatory BP	Age at baseline, change in BMI, γ-glutamyl-transferase, urinary Na:K, anti-HTN drugs, smoking (no change, quit, acquired), oral contraceptive use	BCd – DBP among men never on anti-HTN drugs BCd + SBP, DBP among premenopausal women Longitudinal BCd + DBP among women UCd – SBP, DBP among men never on anti-HTN drugs UCd – 24 hr SBP among perimenopausal and postmenopausal women	Cross-sectional (CadmiBel 1985–1989 and Prospective PheeCad compared 1991–1995 with 1985–1989): A C B/C B	Staessen et al. 2000
Croatian men 20–54 years of age, andrology clinic; excluded occupationally exposed, HTN-treated subjects, renal or other disease that could affect BP; median BCd, 0.83 μg/L; *n* = 154	BCd	SBP, DBP	BMI, blood lead, alcohol, blood copper, smoking	BCd o SBP BCd o DBP	Case–control: B C B B	Telisman et al. 2001

Abbreviations: BMI, body mass index; HDL, high-density lipoprotein; KHANES, Korean National Health and Nutrition Examination Survey; MBP, mean blood pressure; nM/3, nanomolar/3-hr urine; NHANES, National Health and Nutrition Examination Survey; T, tertile.

a Key findings: +, significant positive association; −, significant inverse association; o, null association.

b WOE codes: 1, association; 2, environmental equivalence; 3, population equivalence; 4, bias.
